# Somatic copy number alterations detected by ultra-deep targeted sequencing predict prognosis in oral cavity squamous cell carcinoma

**DOI:** 10.18632/oncotarget.4336

**Published:** 2015-06-02

**Authors:** Chien-Hua Peng, Chun-Ta Liao, Ka-Pou Ng, An-Shun Tai, Shih-Chi Peng, Jen-Pao Yeh, Shu-Jen Chen, Kuo-Chien Tsao, Tzu-Chen Yen, Wen-Ping Hsieh

**Affiliations:** ^1^ Departments of Resource Center for Clinical Research, Chang Gung Memorial Hospital, Taoyuan, Taiwan, R.O.C.; ^2^ Otorhinolaryngology, Head and Neck Surgery, Chang Gung Memorial Hospital, Taoyuan, Taiwan, R.O.C.; ^3^ Head and Neck Oncology Group, Chang Gung Memorial Hospital, Taoyuan, Taiwan, R.O.C.; ^4^ Institute of Statistics, National Tsing Hua University, Hsinchu, Taiwan, R.O.C.; ^5^ Department of Nuclear Medicine and Molecular Imaging Center, Chang Gung Memorial Hospital, Taoyuan, Taiwan, R.O.C.; ^6^ Department of Biomedical Sciences, School of Medicine, Chang Gung University, Taoyuan, Taiwan, R.O.C.; ^7^ Medical Biotechnology and Laboratory Science, Research Center for Emerging Viral Infections, Chang Gung Memorial Hospital, Taoyuan, Taiwan, R.O.C.; ^8^ Laboratory Medicine, Chang Gung Memorial Hospital, Taoyuan, Taiwan, R.O.C.

**Keywords:** ultradeep-targeted sequencing, oral cavity squamous cell carcinoma, copy number alteration, biomarker, clinically actionable genes

## Abstract

**Background:**

Ultra-deep targeted sequencing (UDT-Seq) has advanced our knowledge on the incidence and functional significance of somatic mutations. However, the utility of UDT-Seq in detecting copy number alterations (CNAs) remains unclear. With the goal of improving molecular prognostication and identifying new therapeutic targets, we designed this study to assess whether UDT-Seq may be useful for detecting CNA in oral cavity squamous cell carcinoma (OSCC).

**Methods:**

We sequenced a panel of clinically actionable cancer mutations in 310 formalin-fixed paraffin-embedded OSCC specimens. A linear model was developed to overcome uneven coverage across target regions and multiple samples. The 5-year rates of secondary primary tumors, local recurrence, neck recurrence, distant metastases, and survival served as the outcome measures. We confirmed the prognostic significance of the CNA signatures in an independent sample of 105 primary OSCC specimens.

**Results:**

The CNA burden across 10 targeted genes was found to predict prognosis in two independent cohorts. FGFR1 and PIK3CAamplifications were associated with prognosis independent of clinical risk factors. Genes exhibiting CNA were clustered in the proteoglycan metabolism, the *FOXO* signaling, and the *PI3K-AKT* signaling pathways, for which targeted drugs are already available or currently under development.

**Conclusions:**

UDT-Seq is clinically useful to identify CNA, which significantly improve the prognostic information provided by traditional clinicopathological risk factors in OSCC patients.

## INTRODUCTION

Oral cavity squamous cell carcinoma (OSCC) is a leading cause of morbidity and mortality. OSCC is one of the 10 most common cancers worldwide, with most cases being observed in Asia (mainly because of betel quid chewing) [1]. The survival rates of OSCC remain suboptimal, largely because of delays in diagnosis leading to advanced disease. Risky oral habits for OSCC, including cigarette smoking, alcohol consumption, and betel quid chewing, can cause cumulative genetic changes, genomic aberrations, and widespread genomic instability [2]. Therefore, emerging OSCC genomic data hold great promise for predicting prognosis and providing a basis for the development of targeted therapies [3].

Somatic copy number alterations (CNAs) are widespread in cancer genomes and may lead to oncogene activation and/or tumor suppressor gene inactivation in several malignancies [4]. In addition to the common mutations in TP53, NOTCH1, CASP8, FAT1, CDKN2A, HRAS, and USP9X [2, 5, 6], OSCC also develops through the accumulation of multiple CNA events [5, 7-9]. Amplifications in 3q, 5p, 7p, 8q, 11q, and 20q and deletions in 3p, 8p, 9p, and 18q have been observed in most OSCC studies. In general, these CNAs can either result in altered gene dosage or disrupt intragenetic regions. Although previous studies have shown that CNAs can predict prognosis in solid malignancies [5, 8, 10-12], the clinical significance of CNAs in OSCC remains unclear and requires thorough investigation in large clinical cohorts [9]. In this scenario, determining how CNA contributes to clinical outcomes in patients with OSCC is a critical question and technical improvements in genomic methods are crucial to answering it.

The recent availability of ultra-deep targeted sequencing (UDT-Seq) has allowed increasing the depth of sequence coverage to greater than 2000×. Crucially, the ultra-deep coverage allows the highly sensitive detection of DNA sequence changes, even in small subclones. Most of the genes included in commercially available UDT-Seq panels are clinically actionable genes that are frequently mutated in numerous cancer types. Moreover, such genetic changes are clinically actionable by currently available drugs or new molecules under clinical development. This would ultimately facilitate the clinical translation of genetic data from bench to bedside. The sooner genetic alterations are identified, the sooner patients can be transferred into a clinical setting for treatment selection. Because of the low cost and high sensitivity of UDT-Seq, this therapeutically targetable cancer gene panel is increasingly being used in clinical laboratories for treatment selection [13, 14]. Certain clinically actionable genes contain single nucleotide variants (SNVs), whereas others contain CNAs (e.g., EGFR and BRAF) [6, 15]. However, beyond use in identifying SNVs, the potential usefulness of UDT-Seq for detecting CNA remains unclear. How to reliably detect CNAs by using UDT-Seq data remains open to discussion. In this study, we prioritized identifying CNAs in the actionable genes targeted by UDT-Seq. The purpose of this study was to expand the use of UDT-Seq from SNV detection to CNV discovery, and examine the feasibility of UDT-Seq for molecular prognostication and identification of potential therapeutic targets in OSCC.

Currently, computational methods of CNA analysis have been developed for whole exome sequencing (WES). However, various methods require paired control samples [16, 17] and are applicable with small sample sizes only [16, 18, 19]. Thus, these established methods cannot be applied to large samples (like vast collections of tumor specimens), especially in the absence of paired controls. In addition, when control sample size is much smaller than tumor sample size, the PCA/SVD normalization algorithms tend to falsely exclude copy number variations between tumor and normal samples; instead, such methodology captures the internal variation structure of copy number data among the predominant tumor group [20, 21]. PCA projections are influenced by uneven sample group size [22]. Because the UDT-Seq-targeted regions covered in this study are much sparser and smaller than those covered by WES, the small number of targeted regions is insufficient to reliably estimate parameters in hidden Markov model-based inference [23]. Other currently available CNA tools display optimal performances only for the detection of rare CNA (i.e., those with a frequency of <1% in the patient population) [18, 20, 23, 24]. The recently published ONCOCNV considers normalization with multiple factors [25]. However, the ONCOCNV tool only allows analyzing multiple amplicons per gene, making CNA assessment inappropriate for mutation hotspots containing a single amplicon.

To circumvent these issues, we developed a linear model that overcomes the uneven coverage across target regions with a set of normal controls and can correct systematic biases among multiple samples. Herein, 310 formalin-fixed paraffin-embedded (FFPE) tissue specimens from OSCC resections were analyzed for CNA by using UDT-Seq. Because genetic variants may explain the variable clinical trajectories of OSCC patients who share similar traditional risk factors, molecular stratification and targeted therapies are urgently needed. In this article, we extend the conventional utility of UDT-Seq in SNV detection and demonstrate that high-depth UDT-Seq is also clinically useful for identifying CNA. The use of this low-cost, scalable strategy allows the analysis of numerous samples, ensuring adequate statistical power for the detection of significant relationships between CNA and clinical outcomes. The CNAs identified as significantly associated with prognosis were independently confirmed in a validation set comprising 105 additional primary OSCC samples.

## RESULTS

### Sample characteristics

This study comprised 310 patients with stage III or IV OSCC, most of whom (approximately 95%) were male. Risky oral habits were reported by 94% of the participants. Specifically, 82% of the participants were betel quid chewers. All the participants received follow-up examinations for at least 36 months. During the follow-up period, 193 patients (62.26%) died and 57 (18.39%) developed second primary tumors. Tumor relapse were observed in 53.23% of the samples, including local recurrence (21.93%), neck recurrence (27.1%), and distant metastases (26.77%).

Initial UDT-Seq reads were mapped against the hg19 human reference genome by using the built-in software of the sequencer. The mean sequence coverage was 2443-fold, with 95% of the samples covered at > 1500-fold. This coverage was markedly higher than that of general targeted sequencing. The degree of coverage uniformity for each region was also sufficiently high. The mean coefficient of variance for all of the targeted regions was 0.02. Because of the ultra-deep sequence coverage, the differential CNA signals between tumor and normal samples are more apparent and can more reliably be detected. The profile of read depth across the amplicons was highly reproducible among the controls (average pairwise correlation = 0.941). As shown in a previous study [26], the difference in normalized read depth of an amplicon between tumor and reference samples is correlated to the extent of chromosomal copy number changes.

### Detection of copy number alteration

**Figure 1:**
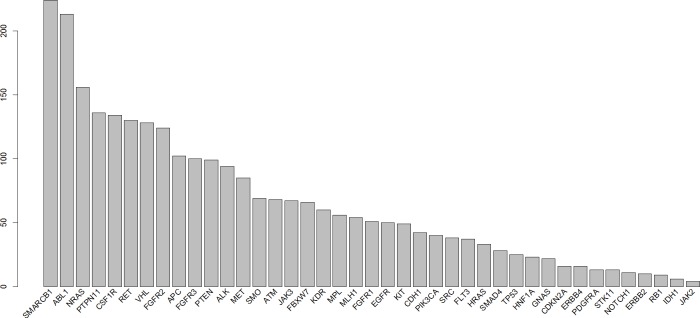
Frequency of copy number amplifications in different genes Each bar indicates the number of samples with copy number amplifications of each gene. The x-axis is sorted using the total frequency of CNA events.

**Figure 2:**
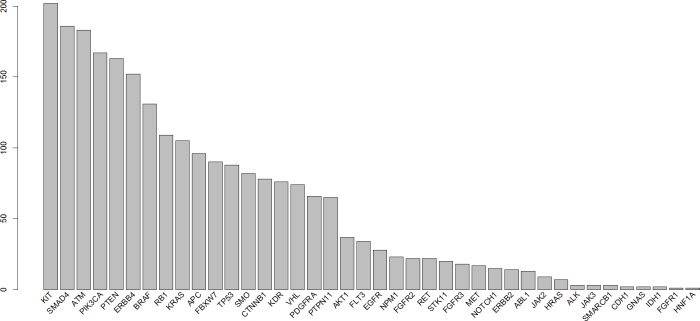
Frequency of copy number deletions in different genes Each bar indicates the number of samples with copy number deletions of each gene. The x-axis is sorted using the total frequency of CNA events.

**Figure 3:**
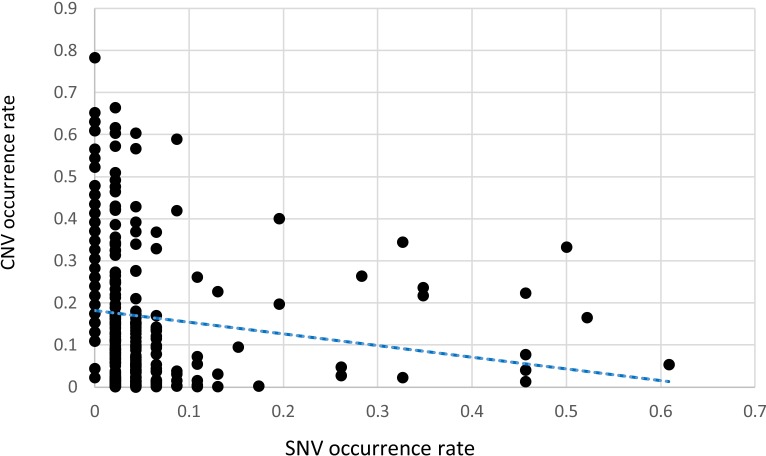
A scatter gram and regression line depicting the relationship between SNVs and CNAs in OSCC specimens The x-axis indicates the fraction of the 46 targeted genes harboring SNV, whereas the fraction of the 46 targeted genes harboring CNAs is depicted on the y-axis. Each point indicates a tumor specimen. In general, CNA were more common than SNV in the targeted genes. The regression line indicates an inverse relationship between the occurrence of SNVs and CNAs.

CNA were determined using the linear model described in the Methods section with a γ of 1.5. All of the examined regions were classified into one of three categories: amplification, deletion, or normal. Most of the targeted regions were found to exhibit CNA in the OSCC samples. However, approximately 50% of the identified CNAs were present in less than 20% of the samples. Specifically, among the 46 targeted genes, 26 genes exhibited CNA with a frequency exceeding 20%. Figures 1 and 2 depict the frequency of copy number amplifications and deletions, respectively. The figures are sorted according to the total number of samples classified as harboring CNAs in the targeted genes. Common amplifications shared by at least 30% of the patients were identified in only 13 genes. Similarly, common deletions occurring in at least 30% of the patients were observed in only 12 genes. According to the number of SNVs reported by the Torrent Variant Caller, we noticed that OSCC samples were characterized by more CNAs than SNVs (Supplementary Table S5). Furthermore, the samples characterized by a higher number of CNAs tended to have a lower number of nonsynonymous SNVs (Figure 3). For each sample, we calculated the SNV occurrence rate as the fraction of the targeted genes harboring SNVs in a particular sample. The CNA occurrence rate was also calculated for an individual sample as the fraction of the targeted genes exhibiting copy number changes. The highest CNA occurrence rate was observed in samples with a SNV occurrence rate of less than 0.06. By contrast, samples with a SNV occurrence rate exceeding 0.1 generally exhibited a CNA rate of less than 0.3. Only 6% of all mutational events were characterized by the presence of CNA and SNV in the same gene. All of the remaining mutations were either isolated CNA or SNV. Therefore, for each target region, the samples harboring CNAs were generally distinct from those carrying SNVs. Such different mutational spectra indicate the existence of different patterns of genetic aberrations in OSCC pathogenesis.

### Validation of copy number alterations

In an effort to validate our CNA calling method, we first assessed the consistency of CNA calls between amplicons covering the same gene. Because CNA size may range from 1 kilobase to several megabases, the two breakpoints of any given CNA segment can fall into an intergenic or intragenic region. When one breakpoint falls into a coding region, the whole CNA segment would encompass some entire genes and one incomplete gene region. Therefore, a CNA event occurring only in a part of a gene may be observed in this study because of the highly discrete nature of amplicon sequencing in UDT-Seq. Considering this, we therefore assessed the consistency of CNA calls between amplicons within a gene using a consistency score. Such score reflects the fraction of copy number-altered amplicons harboring the same amplification/deletion status of the neighboring amplicons within the same gene. The average consistency score among all multi-amplicon genes in all samples was 0.92. More specifically, on average, 92% of all copy number-altered amplicons in a gene were mutually adjacent and exhibited consistent CNA calls.

Second, to evaluate the accuracy of the CNA calling method, we compared the CNA events of the 54 samples for which high-density SNP array profiles were available (Gene Expression Omnibus database accession number: GSE25103). Consequently, the proposed method yielded favorable average accuracy of 88% when we used the CNA results of the SNP array as a standard reference. Because most of the CNA events detected using UDT-Seq were confirmed by the SNP array, we believe that our sequencing approach yielded robust data.

To further validate our UDT-Seq approach for CNA assessment, we utilized a replication panel comprising 105 OSCC specimens. In this cohort, 91% of subjects had risky oral habits and 81% were betel quid chewers. We found that 80.71% of the CNA identified in the first cohort were consistently present in the replication sample, supporting the robustness of our approach. Notably, the frequency of common CNA (identified in more than 30% of the samples) was similar in the discovery and replication cohorts. Consistently common CNA amplifications were observed for the following genes: MET, ABL1, NRAS, FGFR2, FGFR3, CSF1R, VHL, and RET, whereas common CNA deletions involved SMAD4, ATM, PTEN, ERBB4, RB1, APC, FBXW7, and TP53.

### Association between CNA and clinical outcomes

**Figure 4:**
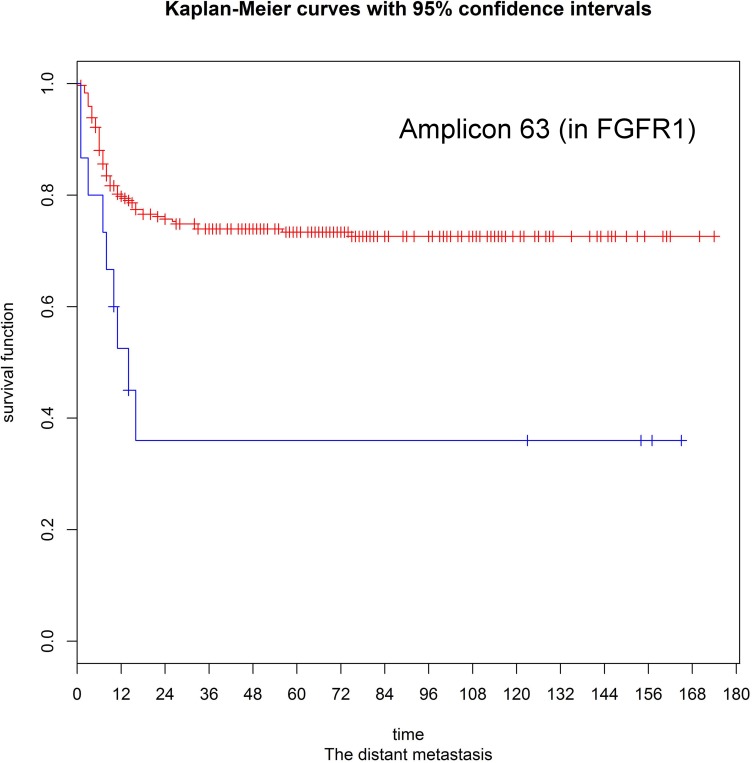
Kaplan-Meier estimates for distant metastases according to the presence or absence of FGFR1 amplifications (log-rank test, *p* = 0.001)

Patients were divided into three subgroups according to their CNA status (e.g., amplification, deletion, or normal) at each target region. We used the log-rank test to assess the associations between clinical outcomes and the presence of CNA events. The resulting *p* values were corrected for multiple testing by using the false discovery rate (FDR) procedure to obtain a maximum FDR of 5%. Significant associations were evident for different CNA events (Supplementary Tables S2 and S3). For example, copy number amplifications in PIK3CA were associated with an increased risk of local recurrence (hazard ratio [HR] = 2.33, *p* = 0.006), as well as lower disease-free (HR = 1.987, *p* = 0.0008), disease-specific (HR = 1.95, *p* = 0.002), and overall (HR = 17, *p* = 0.007) survival rates. Patients with amplifications in the fibroblast growth factor receptor genes (FGFR1, FGFR2, and FGFR3) had a higher risk of distant metastases (*p* = 0.001, 0.013, and 0.0003, respectively). Copy number amplification in the ATM gene was associated with neck control (HR = 24.16, *p* = 0.002) and disease-free (HR = 14.10, *p* = 0.009), disease-specific (HR = 12.8, *p* = 0.012), and overall survival rates (HR = 11.89 and *p* = 0.015). We also observed that deletions in the APC and SMAD4 genes were significantly associated with neck control (HR = 9.742 and 16. 95, *p* = 0.0001 and 0.00001, respectively) and disease-free (HR = 5.702 and 13.23, *p* = 0.003 and 0.00001, respectively), disease-specific (HR = 6.071 and 5.42, *p* = 0.002 and 0.0002, respectively), and overall survival rates (HR = 5.794 and 5.114, *p* = 0.002 and 0.0003). Besides, copy number deletions within the RB1 gene were significantly associated with local recurrence (HR = 4.998, *p* = 0.001), neck control (HR = 2.451, *p* = 0.011), and disease-free (HR = 3.021, *p* = 0.004), disease-specific (HR = 3.6, *p* = 0.001), and overall survival rates (HR = 2.558, *p* = 0.015). We also identified copy number deletions in the tumor suppressor PTEN as significantly associated with poor disease-free (HR = 31.15, *p* = 0.00009), disease-specific (HR = 31.53, *p* = 0.00009), and overall survivals (HR = 27.21, *p* = 0.0001). Other significant relationships are listed in Supplementary Table S3. To validate the associations identified in the discovery panel, we sought to confirm the results using a replication panel. The successfully replicated associations are summarized in Tables 1 and 2. With regard to the three fibroblast growth factor receptors, only FGFR1 amplification retained its significant predictive value for distant metastasis in the replication panel (Figure 4). Similarly, CNAs identified in the PIK3CA, RB1, ATM, SMAD4, and PTEN genes were consistently associated with different clinical outcomes (Supplementary Figures S1 and S2). Of all the validated associations, we found amplifications and deletions occurring in the RB1 gene exhibited a significant prognostic impact. In both the discovery and validation sets, copy number amplifications of RB1 were rare, even though deletions were frequent.

**Table 1:** Validated associations between copy number amplifications and clinical outcomes

Clinical outcome	CNA-harboring gene	Hazard ratio	*P* value
Local recurrence	PIK3CA	2.332341	0.006
Distant metastases	FGFR1	3.043851	0.001
Distant metastases	SMO	8.034381	0.003
Distant metastases	RB1	9.043599	0.002
Distant metastases	RET	13.32504	0.011
Disease-free survival	PIK3CA	1.987948	0.0008
Disease-free survival	ATM	14.10226	0.009
Disease-free survival	STK11	31.09854	0.0009
Disease-free survival	RET	14.37666	0.009
Disease-specific survival	PIK3CA	1.945889	0.002
Disease-specific survival	ATM	12.80487	0.012
Disease-specific survival	STK11	31.24429	0.0009
Overall survival	PIK3CA	1.69732	0.007
Overall survival	ATM	11.89914	0.015
Overall survival	CDH1	3.323677	0.008
Overall survival	STK11	27.2731	0.001

**Table 2:** Validated associations between copy number deletions and clinical outcomes

Clinical outcome	CNA-harboring gene	Hazard ratio	*P* value
Distant metastases	PTEN	66.15871	0.0001
Distant metastases	RB1	2.270029	0.011
Distant metastases	TP53	2.025808	0.024
Distant metastases	SMAD4	11.59482	0.00003
Disease-free survival	APC	5.702536	0.003
Disease-free survival	PTEN	31.15534	0.0009
Disease-free survival	RB1	3.021541	0.004
Disease-free survival	SMAD4	5.556417	0.003
Disease-specific survival	APC	6.071924	0.002
Disease-specific survival	MET	5.472896	0.017
Disease-specific survival	PTEN	31.53222	0.0009
Disease-specific survival	RB1	3.600392	0.001
Disease-specific survival	SMAD4	5.426748	0.0002
Overall survival	APC	5.794595	0.002
Overall survival	MET	5.214931	0.020
Overall survival	PTEN	27.2186	0.001
Overall survival	SMAD4	5.144378	0.0003

To assess the independent contribution of the identified CNA to clinical outcomes, we constructed multivariate Cox proportional hazard models using conventional clinicopathological risk factors and the CNA events listed in Tables 1 and 2 as covariates. The conventional risk factors included in the model were pathological stage, pathologically positive nodes, pathological T status, and extracapsular spread (ECS) [27-30]. The results indicated that pathological stage was an independent predictor of neck control (HR = 2.26, *p* = 0.009). With the only exception of neck control, CNA events retained their independent prognostic significance in the multivariate analysis. Specifically, amplification in FGFR1 was independently associated with the rates of both local recurrence (HR = 2.12, *p* = 0.011) and distant metastases (HR = 2.28, *p* = 0.006; Table 3). PIK3CA amplification also reached significance for the prediction of local recurrence (HR = 2.63, *p* = 0.007) and disease-free (HR = 2.29, *p* = 0.025), disease-specific (HR = 2.26, *p* = 0.019), and overall survival rates (HR = 2.12, *p* = 0.002).

**Table 3:** Multivariate analysis of clinicopathological traits and CNA events (validated in the replication panel) for the prediction of clinical outcomes

Clinical outcome	Predictor	Hazard ratio	*P* value
Second primary tumors	SMAD4 deletion	1.81	0.016
Second primary tumors	STK11 amplification	2.09	0.041
Second primary tumors	TP53 deletion	1.72	0.025
Second primary tumors	ATM amplification	1.72	0.041
Second primary tumors	pN2c	2.08	0.05
Local recurrence	Pathological tumor stage	1.4	0.011
Local recurrence	RB1	1.52	0.049
Local recurrence	FGFR1 amplification	2.12	0.011
Local recurrence	PIK3CA amplification	2.63	0.007
Neck control	Pathological stage	2.26	0.009
Distant metastases	Extracapsular spread	3.39	0.000013
Distant metastases	Pathological tumor stage	1.41	0.005
Distant metastases	FGFR1 amplification	2.28	0.006
Disease-free survival	Extracapsular spread	1.8	0.0004
Disease-free survival	Pathological tumor stage	1.36	0.0002
Disease-free survival	PIK3CA amplification	2.29	0.025
Disease-specific survival	Extracapsular spread	2.14	0.00005
Disease-specific survival	Pathological tumor stage	2.93	0.0003
Disease-specific survival	PIK3CA amplification	2.26	0.019
Overall survival	Extracapsular spread	1.74	0.0002
Overall survival	Pathological tumor stage	1.38	0.00005
Overall survival	PIK3CA amplification	2.12	0.002

1 Outcomes independently predicted by validated CNA are marked in bold.

Because CNA were significant independent predictors of outcomes even after allowance for traditional risk factors in multivariate models, we used real-time quantitative polymerase chain reaction (qPCR) to further validate our predicted copy number status. There were a total of 140 remaining DNA samples available for qPCR analyses, although their quantity was limited. Therefore, we selected prognostically important genes, PIK3CA and FGFR1, to be validated using qPCR because the CNAs in both genes were independently associated with multiple prognostic outcomes in the multivariate model. The experimental validation showed a mean accuracy of 85%. Accuracy was defined as the fraction of samples whose copy number (CN) status were replicated in the qPCR results. Similar to PIK3CA amplification, PTEN deletion may constitutively activate the PI3K signaling cascade [31]. PTEN deletions were common in our study and its prognostic impact was successfully replicated in the independent cohort. We thus decided to confirm the CN status of PTEN using qPCR. The result revealed that 80% of its CN status were validated. Common SNVs in TP53 have been previously associated with OSCC. The clinical significance of SNVs in TP53 is already well known. However, TP53 deletions were also observed in the current study. Thus, we further validated TP53 deletion. The results of validation experiments indicated a mean accuracy of 87%. By contrast, several CNA associations involving amplifications in EGFR, RET, and ABL1 and deletion in BRAF were not significant after FDR correction but were still detected in the independent cohort. To confirm these CNA events, the validation of CNA in EGFR, RET, and BRAF by qPCR indicated a mean accuracy of 84%. As for ABL1, the accuracy was 70%. It should be emphasized that not all true CNAs could be detected using qPCR method. The sensitivity of qPCR decreases in presence of low-copy number variations. Thus, the accuracy results may be only conservative estimates.

To further investigate whether the knowledge of CNA could provide additional predictive power when combined with clinical risk factors, we assessed the predictive power of the individual integrated models reported in Table 3 and their corresponding clinical-factors-only models. For each prognostic outcome, the corresponding clinical-factors-only model included the clinical variables independently associated with the outcome of interest. Accordingly, we estimated the predictive power using the concordance index (c-index) for each prognostic outcome separately. The c-index was calculated using the R package survcomp to measure the probability of concordance between predicted and observed responses [32]. A c-index exceeding 0.5 implies good prediction ability, whereas a c-index equal to 0.5 indicates random findings. When the c-index is less than 0.5, the predicted response is reversed. The significance of a difference in c-index between the integrated and clinical-factors-only models was calculated as described by Haibe-Kains et al. [32]. Consequently, a c-index increase was observed in all outcomes when the CNA markers were added to the clinical-factors-only model (Table 4). However, for the second primary development, the additional predictive power of the CNA markers from 0.67 to 0.71 was nonsignificant (*p* = 0.073). Notably, the c-index significantly increased from 0.63 to 0.87 when PIK3CA amplification was added to a model including extracapsular spread and pathological tumor stage for predicting disease-specific survival (*p* = 0.00006). The same promising improvement in the predictive power was observed when PIK3CA amplification was added to the model for disease-free survival (c-index: 0.66–0.85, *p* = 0.0003).

**Table 4:** Comparison of predictive power of a clinical-factors-only model versus an integrated model comprising CNA and clinical risk factors

Clinical outcome	C-index		*P* value
	Using clinical-factors-only	Using both of CNA markers and clinical risk factors	
Second primary tumors	0.67	0.71	0.073
Local recurrence	0.65	0.77	0.0032
Distance metastasis	0.72	0.82	0.0027
Disease-free survival	0.66	0.85	0.0003
Disease-specific survival	0.63	0.87	0.00006
Overall survival	0.66	0.73	0.048

### Association between CNA and HPV status

In addition to risky oral habits, human papillomavirus (HPV) infections have been linked to oral carcinogenesis. Because HPV 16 and 18 have been associated with an increased risk of OSCC [33-35], we investigated whether an association exists between CNA and HPV infection status. Notably, PIK3CA amplification has been linked to HPV infection in oropharyngeal squamous cell carcinoma [36]. In accordance with these data, we found a significant association between copy number amplifications in PIK3CA and HPV 16/18 infection (*p* = 0.004). These findings support both the validity of previous data and the robustness of our UDT-Seq approach for determining CNA. We also observed that amplifications in ATM (*p* = 0.015), CDH1 (*p* = 0.029), and NOTCH1 (*p* = 0.001) and deletions in TP53 (*p* = 0.04) were significantly more frequent in HPV 16/18-positive OSCC patients.

## DISCUSSION

In this study of primary OSCC tumor specimens, we utilized UDT-Seq to identify CNA in cancer genes. Two normalization steps based on linear models were applied to overcome both coverage biases across target regions and batch effects across samples. The time complexity of our linear model is bounded by linear time, ultimately allowing its use in large datasets. To our knowledge, no other analytical method has been described for using UDT-Seq to detect amplifications or deletions in a single amplicon.

Because standard clinicopathological risk factors have limited ability to predict outcomes in the heterogeneous population of patients with OSCC, identifying new prognostic biomarkers is an urgent task. The prognostic CNAs identified in our study were consistent with those reported previously (Supplementary Table S4), supporting the methodological validity of our genotyping approach. For example, PIK3CA amplification was observed as ranging from 9% to 66% for OSCC [31], and FGFR1 amplification was found in 33% of patients with OSCC [37]. Deletions of PTEN (29%), RB1 (66%), SMAD4 (11%), and TP53 (56%) have been previously reported in independent OSCC cohorts [38-41]. Accordingly, the prevalence of CNA in TP53 (28%), FGFR1 (17%), and RB1 (35%) was lower in this study as compared with previous research conducted in Western countries. In turn, deletions in PTEN (53%) and SMAD4 (60%) were more frequently observed compared with previous studies. Such discrepancies may be explained at least in part by ethnic differences in risky oral habits. Betel quid is the main risk factor for OSCC in Eastern countries, whereas cigarette smoking and alcohol consumption are the main causative agents in the West [42, 43]. Hence, the underlying tumorigenic mechanism in OSCC in Asia might differ from that in Western countries.

Because both the CNA amplifications and deletions observed in this study contribute to carcinogenesis in numerous solid malignancies [31, 37, 44-52], we sought to investigate their associations with clinical outcomes in two independent cohorts of patients with OSCC. The independent prognostic associations were successfully replicated (Tables 1 and 2). Thus, we explored the distributions of those prognostic CNA predictors using molecular pathways. Some of the prognostic CNA events identified in our study (e.g., FGFR1 amplification) were found to be distributed in the proteoglycan metabolism pathway, particularly in the heparan sulfate proteoglycan biosynthetic process. Heparan sulfate proteoglycans are involved in numerous tumorigenesis processes, including cell growth, differentiation, and angiogenesis. Because the enzymatic modification of heparan sulfate proteoglycans can dramatically modify tumor cell behavior [53], our data suggest that the heparan sulfate proteoglycan biosynthetic process is a potential therapeutic target for patients with OSCC who have the CNA in this pathway. In addition, other prognostic CNAs also clustered in the PI3K–AKT signaling pathways. The PI3K–AKT signaling pathway comprises key survival factors involved in the control of cell proliferation, apoptosis, and oncogenesis and is hyperactivated in several malignancies [54]. Among the aberrant genes in this pathway, PIK3CA amplification retained its independent prognostic significance for multiple clinical outcomes in the multivariate analysis. PIK3CA amplification constitutively activates PI3K, thereby triggering continuous activation of downstream signaling (e.g., mTOR pathway). Interestingly, PIK3CA amplification has been shown to be correlated with metastatic OSCC [31]. Other CNA events in the PI3K–AKT signaling pathway, such as loss of the PTEN suppressor, could exert the same effect as PIK3CA amplification [31]. Furthermore, we also observed prognostic CNAs accumulated in the FOXO signaling cascade, which is downstream to the PI3K–AKT pathway. The FOXO family of transcription factors are master regulators of cell growth, *proliferation*, *differentiation*, apoptosis, and autophagy [54]. Notably, phosphorylation of FOXO by AKT inhibits their transcriptional functions, causing cell survival and proliferation [54]. Hence, in the absence of PTEN-induced downregulation of AKT, FOXO transcription factors are constitutively phosphorylated, thereby inhibiting the activation of the programmed cell death pathway. However, copy number deletions in PTEN were common (52%) in the current study. FOXO signaling may be constitutively blocked in patients with OSCC who have PTEN deletion. In light of these findings, the repression of the PI3K–AKT pathway (e.g., by PI3K inhibitors) and induction of FOXO transcription factors (e.g., by AKT inhibitors that suppress FOXO phosphorylation) may be considered as attractive therapeutic targets for patients with OSCC who have the CNAs in those pathways. Indeed, inhibitors of the PI3K–AKT pathway are currently in various stages of development in clinical trials [55-59]. Our results may thus provide a basis for genomic-driven clinical trials in OSCC patients aimed at developing targeted drugs.

However, our current findings should be interpreted within the context of some limitations. Specifically, betel quid chewing is endemic in the study area and caution should be exercised when generalizing the results to other populations. Nonetheless, using UDT-Seq to determine CNA may facilitate the molecular classification of OSCC into prognostically relevant subtypes and provide new insights into different oncogenic pathways. Although UDT-Seq could miss large CNA events, it can be clinically useful for the rapid identification of CNA patterns in OSCC. Furthermore, the UDT-Seq assay is capable of evaluating multiple genes in target pathways (e.g., FOXO signaling) in a simultaneous manner, ultimately reducing costs and making it appropriate for clinical applications.

In conclusion, the proposed linear model of CNA discovery is feasible for analyzing UDT-Seq data. This is the first study investigating the association of UDT-Seq-identified CNA with clinical outcomes in OSCC specimens. The additional predictive power of the CNA markers was also demonstrated. Importantly, the identified CNA were located in clinically actionable genes that may serve as therapeutic targets. There are two principal routes through which our current results may be translationally relevant. First, identifying the main disturbed pathways (i.e., the proteoglycan metabolism, FOXO and PI3K–AKT signaling) can aid in identifying novel therapeutic strategies. Second, CNA-based biomarkers can improve clinical outcome prediction and the monitoring of disease progression and treatment response. Future studies should provide proof of principle that combining these molecular classifiers with traditional clinicopathological risk factors could improve prediction accuracy.

## MATERIALS AND METHODS

### Ethics statement

This study was performed according to national and international guidelines and was approved by the Research Ethics Board of Chang Gung Memorial Hospital (CGMH 101-4457B). The requirement for patient consent was waived because of the retrospective nature of the study.

### Study participants

We retrospectively reviewed the medical records of 310 patients with previously untreated primary OSCC who were referred for radical tumor excision and neck dissection between 1996 and 2009. Fourteen people without malignancies served as controls. All of the participants underwent an extensive evaluation before primary surgery. Patients were staged according to the 2010 American Joint Committee on Cancer staging criteria. The clinicopathological traits and follow-up data of each patient were obtained from general practice records. The following variables of all participants were collected: age at OSCC onset, sex, risky oral habits (cigarette smoking, alcohol drinking, betel quid chewing), ECS, and follow-up length.

In our hospital, collecting sufficient normal oral tissue from patients with OSCC is challenging. However, basal copy number information could also be obtained from the general population. Blood lymphocytes from healthy humans have also been used as a diploid standard in OSCC [60]. Therefore, we used DNA from the peripheral blood mononuclear cells (PBMC) of 16 healthy donors to generate basal sequencing data. These PBMC samples were obtained from regular healthy check-up people at Chang Gung Memorial Hospital. Genomic DNA was prepared using the Qiagen Mini Kit (Qiagen, Hilden, Germany). DNA concentration was determined using the Qubit fluorometer (Invitrogen, Calsbad, California, USA). DNA integrity was analyzed using the Agilent Bioanalyzer 2100. The same sequencing procedure was conducted for both tumor and normal samples (see below).

### Surgery and adjuvant therapy

The primary tumors were excised with safety margins of 1 cm or more (both peripheral and deep margins). Level I–V neck dissections were performed in patients with cN+ disease, whereas cN– patients received level I–III neck dissections. Postoperative radiotherapy (60 Gy) was administered to patients exhibiting pathological risk factors. The radiation field included the entire tumor bed area (with 1–2 cm margins) and regional lymphatics. Concomitant chemoradiation (66 Gy) with cisplatin-based regimens were administered to patients with ECS, multiple lymph node metastases, and positive margins.

### Ultra-deep targeted sequencing

The Ion Torrent AmpliSeq™ Cancer Panel (Life Technologies, Carlsbad, California, USA) allows the enriching of up to thousands of genomic targets from 10 ng of DNA extracted from FFPE samples by using the QIAmp DNA FFPE DNA extraction kit (Qiagen, Hilden, Germany). The AmpliSeq™ panel comprises 189 primer pairs that are designed to amplify the mutation hotspots of 46 oncogenes and tumor suppressor genes (Supplementary Table S1). After 20 initial PCR cycles, the amplicons were ligated with sequencing barcode adapters and then subjected to five additional cycles. Barcoded libraries were produced with 50 ng of amplicons by using the Ion Plus Fragment Library Kit (Life Technologies), and the resulting products were sequenced on an Ion 318 chip.

### CNA detection

In general, the assumption of any read depth of coverage method is that the read density of a target region is roughly proportional to the number of copies in that region. However, the quantitative relationship between the actual copy number and sequence depth may be distorted by the efficiency of multiplex PCR method, which can introduce both region- and sample-specific biases. To address this potential problem, we developed a linear model to normalize sequencing data. Because we examined short-length mutation hotspots, we assumed a consistent copy number within any specific region. The average read depth of each target region was consequently used to indicate the sequence abundance of the corresponding loci. Before normalization, log-transformation was performed to stabilize the variance of the average read depth of each region. Consequently, the variability of each read depth was unrelated to its mean value. The normalization step was aimed at determining the extent of CNA relative to the control samples. To produce normalized signals, the proposed method enables correction for both region- and sample-specific biases through the following steps:

#### Step 1: Assessment of region-specific effects using control samples

We initially reasoned that the PCR efficiency of the target sequences may vary across regions because of guanine cytosine content, size, and sequence complexity. Therefore, we used control samples to generate a reference measure of region-specific variation across the target regions. The availability of a set of control samples is essential for studies without paired matched controls.

Suppose that *y_ij_* is the average read depth of the *i*^th^ region in the *j*^th^ control sample, where *i = l,...,m*, *j = l,...,k*. The region-specific effect in the control samples can be calculated as follows:

(1) $$\log_{2}(y_{ij})=\mu_{N}+\alpha_{i}+\beta_{j}+\varepsilon_{ij}$$

where *μ_N_* represents the overall log control sample mean, *α_i_* is the effect of the *i*^th^ genomic region, *β_j_* is the effect of the *j*^th^ control sample, and *ε_ij_* is the random error of a normal distribution with an expectation of 0 and variance of $\sigma_{N}^{2}$. Because the region profiles denoted by *y_i1_, y_i2_,…,y_ik_* exhibit high similarity among different controls, the estimate αi^ represents the relative sequencing preference among different regions. The control sample mean and the intrinsic region-specific effect, *μ_N_* + *α_i_* may be considered as the basal average read depth of region *i*.

#### Step 2: Correction for region- and sample-specific effects in cancer specimens

In general, PCR efficiency may vary across samples. Such a bias may be due to different DNA concentrations, hybridization temperatures, and batch effects. Therefore, we identified a strategy to minimize this analytical source of confounding. Suppose that *y_ij_* is the average read depth of the *i*^th^ region in the *j*^th^ tumor sample, where *i* = 1,2,…,*m*, and *j*=1,2,…,*l*. After adjustment for region-specific baseline values, the read depth of region *i* in sample *j* can be modeled as:

(2) $$z_{ij}=\mu_{C}+\beta_{j}+\varepsilon_{ij}$$

where $z_{ij}=\log_{2}(y_{ij})-{\hat{\mu }}_{N}-{\hat{\alpha }}_{i}$, *i* = 1,2,…,*m* and *j* = 1,2,…*l*; *μ_C_* indicates the overall tumor sample mean after removing the region effect; *β*_j_ is the effect of the *j*^th^ tumor sample; and *ε_ij_* indicates the random error following a normal distribution with an expectation of 0 and variance $\sigma_{C}^{2}$. After fitting the model with tumor samples, the residual ${\hat{\varepsilon }}_{ij}$ is calculated by removing the estimates of *μ_N_* and *z_ij_* from the adjusted read depth *z_ij_* as follows:

$${\hat{\varepsilon }}_{ij}=z_{ij}-{\hat{\mu }}_{c}-{\hat{\beta }}_{j}=\log_{2}(y_{ij})-\mu_{N}-\alpha_{i}-{\hat{z}}_{ij}=log_{2}\left(\frac{y_{ij}}{2^{{\hat{\mu }}_{N}+{\hat{\alpha }}_{i}+{\hat{z}}_{ij}}}\right)=\log_{2}\left(\frac{y_{ij}}{{\hat{y}}_{ij}}\right)$$

The equation indicates that the residual ${\hat{\varepsilon }}_{ij}$ is the log-ratio between *y_ij_* and ${\hat{y}}_{ij}$, where *y_ij_* is the observed average read depth and ${\hat{y}}_{ij}$ is the corresponding estimate of the expected average read depth for the *i*^th^ region in the *j*^th^ tumor sample. Consequently, the residual ${\hat{\varepsilon }}_{ij}$ reflects the discrepancy between the observed read depth and the expected read depth mean. Accordingly, the relative copy number change of each tumor specimen may be inferred from the residuals. For the residual ${\hat{\varepsilon }}_{ij}$ obtained through the normalization step, we have to determine which deviation from the zero level is sufficient for calling a copy number change. Because the residuals may vary slightly across regions, we scaled down the residuals in each region with their own standard deviations to make the adjusted residuals comparable across different regions. Th process is summarized as follows:

1. For region *i*, the standard deviation *S_i_* is calculated from the residuals ${\hat{\varepsilon }}_{i1,}\varepsilon_{i2},\ldots ,\varepsilon_{il}$.
2. The adjusted residuals are calculated as $\hat{\varepsilon }{'}_{ij}=\frac{{\hat{\varepsilon }}_{ij}}{S_{i}}$.

Notably, we did not adjust the residuals with the region-specific mean because that may have overcorrected the residuals in highly recurrent CNA regions. Instead, the overall mean of all adjusted residuals was used as a reference point. Starting from these assumptions, CNA calls can be determined at the resolution of individual regions. We defined a region as harboring copy number amplifications if the adjusted residual exceeded γ standard deviations above the overall mean, where the standard deviation and mean are calculated from all the adjusted residuals. A copy number deletion was considered to be present if the adjusted residual was less than γ standard deviations below the overall mean. Our approach allowed the determination of CNA calls in each region of tumor specimens. As expected, the higher the γ applied, the fewer false CNAs were included.

### CNA validation by quantitative PCR

CNAs were validated by using TaqMan^®^ Copy Number Assays to perform qPCR. TaqMan Copy Number Assays (Life Technologies) were performed according to the manufacturer instructions. We used PBMC samples from healthy donors as the controls and the qPCR values were normalized to the endogenous control, RNase P. A total of 10 ng genomic DNA from each sample was used as template. The PCR conditions were as follows: an initial denaturation step at 95°C followed by 40 cycles consisting of a 15 sec denaturation at 95°C, a 60 sec annealing and extension step at 60°C. All data were analyzed with the typical comparative 2^−ΔΔCt^ cycle threshold method using the CopyCaller^®^ Software v2.1.

### Statistical analysis for clinical association

The study endpoints included the rates of second primary tumors, local recurrence, neck recurrence, distant metastases, disease-free survival, disease-specific survival, and overall survival. Overall survival and disease-specific survival were calculated from the date of primary surgery to the date of death from any cause and OSCC, respectively. Disease-free survival was measured as the time elapsed from the date of primary surgery to the date of tumor relapse. The rates of local recurrence, neck recurrence and distant metastases were calculated from the date of surgery to the date of local, neck, and distant events, respectively. Survival curves were plotted using the Kaplan-–Meier method and compared using the log-rank test. Multivariate hazard ratios for time-to-event outcomes were calculated using forward stepwise Cox regression models. The association between HPV infection status and CNA in each target region was analyzed using the χ^2^ test. All statistical calculations were performed using R package and the SPSS software package 21.0 for Windows (SPSS Inc., Chicago, Illinois, USA).

#### Consistency score

To calculate the percentage of copy number-altered amplicons that harbored the same CNA status of their neighboring amplicons within the same gene, we formulated a consistency score C, as follows:

$$c=\frac{N_{c}}{N_{a}}$$

where *N_a_* is the number of copy number-altered amplicons within a gene and *N_c_* is the number of copy number-altered amplicons whose CNA status is consistent with their neighboring amplicons.

#### Concordance index

The concordance index indicates the fraction of concordant pairs of patients among all possible pairs. The index can be interpreted as indicating the probability that, given two randomly selected patients, the patient who experiences the event at a later time point had a lower risk of the event [61, 62]. The concordance index was calculated as follows:

$$c-\text{index}=\frac{\sum_{i,j\in P}l_{f(x_{i})<f(x_{j})}}{\left|P\right|}$$

where *f(x_i_)* and *f(x_j_)* are the risk predictions of patients *i* and *j*, respectively, and the indicator function *l_f(xi)<f(xj)_* = 1 if *f(xi)*<*f(xj)* and 0 otherwise. *P* is the set of all possible pairs of patients.

## SUPPLEMENTARY FIGURES AND TABLES




