# Supplementary material for: Somatic copy number alterations detected by ultra-deep targeted sequencing predict prognosis in oral cavity squamous cell carcinoma
Source: Oncotarget. 2015 Jun 2;6(23):19891–906. doi: 10.18632/oncotarget.4336 (PMC4637328; doi:10.18632/oncotarget.4336)
Supplement: Supplementary file 1 [file oncotarget-06-19891-s001.pdf]

Somatic copy number alterations detected by ultra-deep targeted sequencing predict prognosis in oral cavity squamous cell carcinoma

Supplementary Material

The filtering process for SNVs reported by the Torrent Variant Caller.

All of the SNVs reported by the Torrent Variant Caller should satisfy each of the following conditions:

1. Allelic frequency ≧ 0.03.
2. Absence in the database of common variants, as defined by the dbSNP.
3. Absence in the SNP database of the International HapMap Project (general population database).
4. Absence in the SNP database of the 1000 Genomes Project (general population database).
5. Nonsynonymous SNV or SNV occurred in splicing sites.

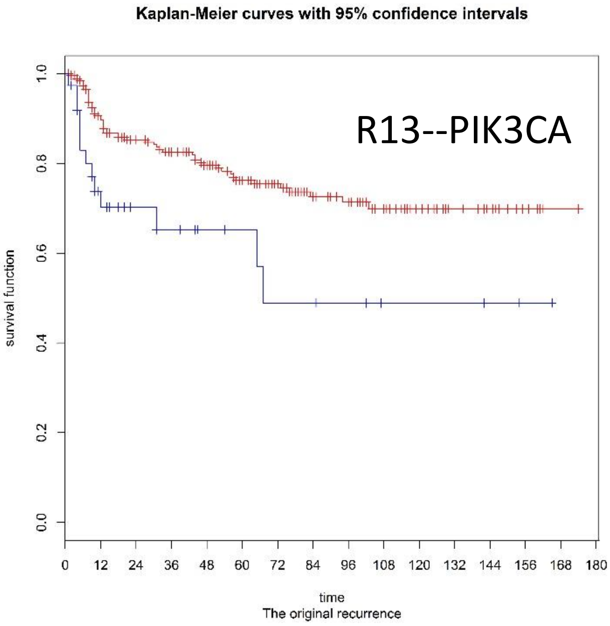
Kaplan-Meier curves with 95% confidence intervals
R13--PIK3CA
survival function
0.0 0.2 0.4 0.6 0.8 1.0
0 12 24 36 48 60 72 84 96 108 120 132 144 156 168 180
time
The original recurrence

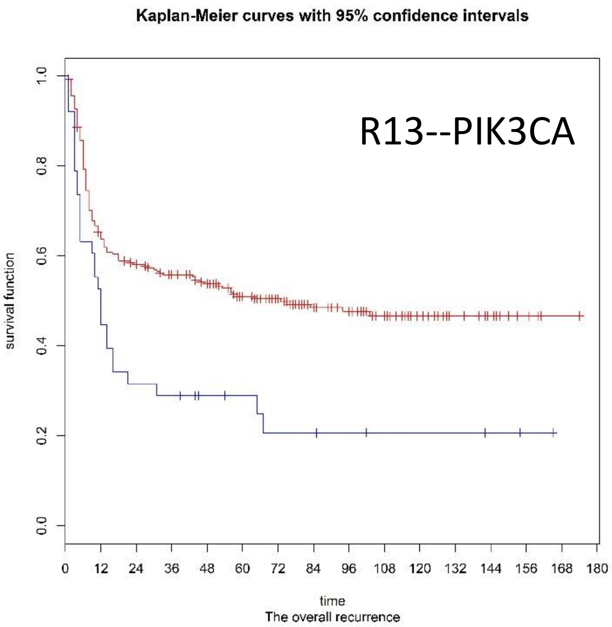
Kaplan-Meier curves with 95% confidence intervals
R13--PIK3CA
survival function
0.0 0.2 0.4 0.6 0.8 1.0
0 12 24 36 48 60 72 84 96 108 120 132 144 156 168 180
time
The overall recurrence

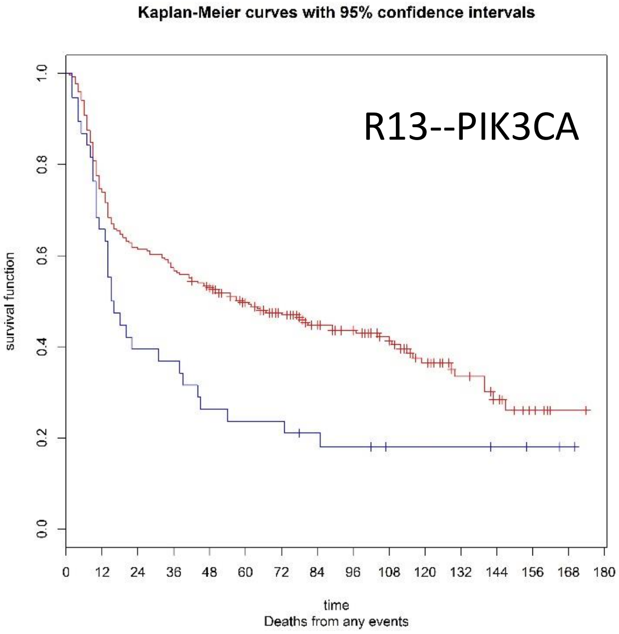
Kaplan-Meier curves with 95% confidence intervals
R13--PIK3CA
survival function
0.0 0.2 0.4 0.6 0.8 1.0
0 12 24 36 48 60 72 84 96 108 120 132 144 156 168 180
time
Deaths from any events

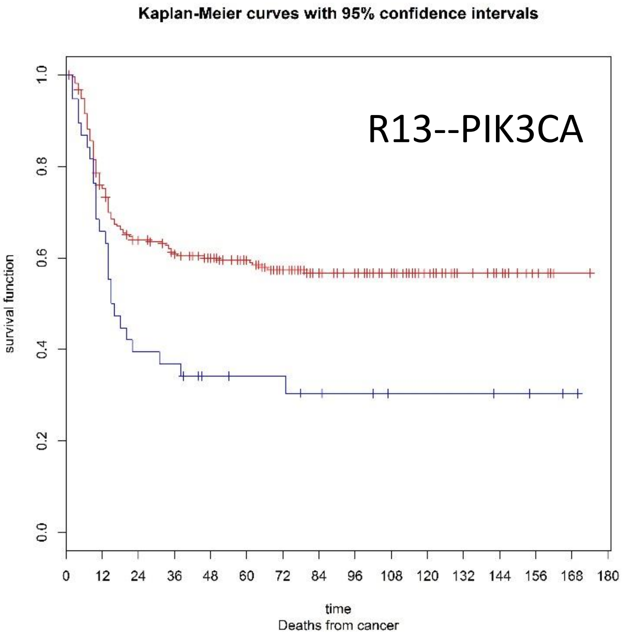
Kaplan-Meier curves with 95% confidence intervals
R13--PIK3CA
survival function
0.0 0.2 0.4 0.6 0.8 1.0
0 12 24 36 48 60 72 84 96 108 120 132 144 156 168 180
time
Deaths from cancer

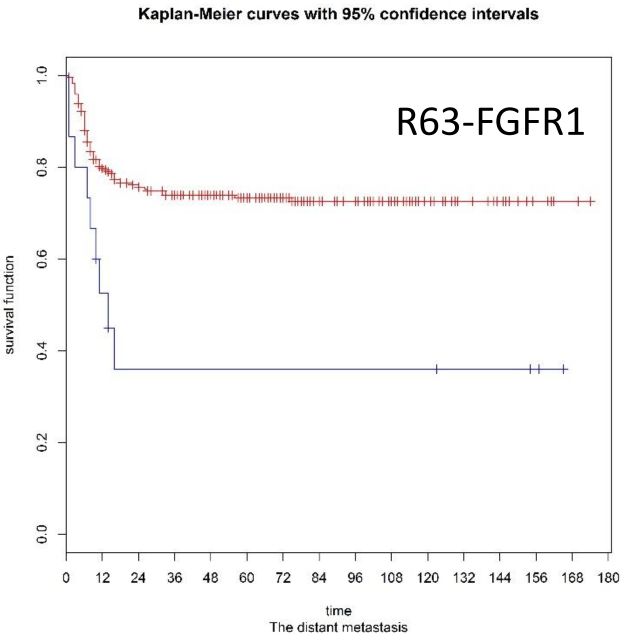
Kaplan-Meier curves with 95% confidence intervals
R63-FGFR1
survival function
0.0 0.2 0.4 0.6 0.8 1.0
0 12 24 36 48 60 72 84 96 108 120 132 144 156 168 180
time
The distant metastasis

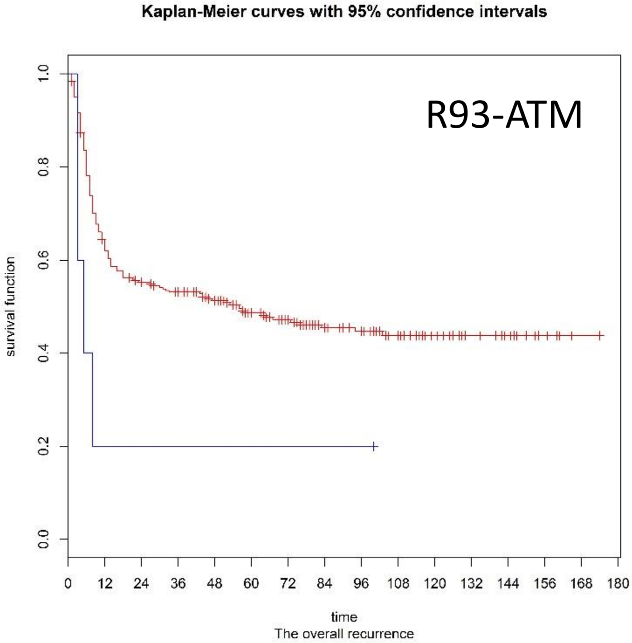
Kaplan-Meier curves with 95% confidence intervals
R93-ATM
survival function
0.0 0.2 0.4 0.6 0.8 1.0
0 12 24 36 48 60 72 84 96 108 120 132 144 156 168 180
time
The overall recurrence

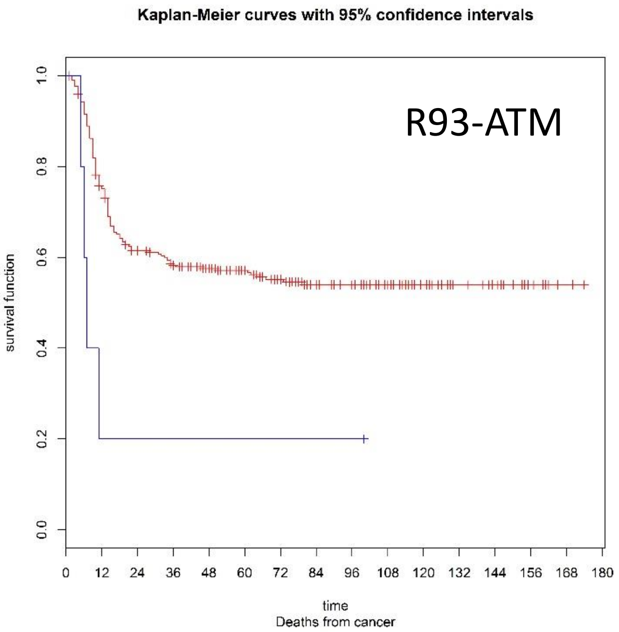
Kaplan-Meier curves with 95% confidence intervals
R93-ATM
survival function
0.0
0.2
0.4
0.6
0.8
1.0
0 12 24 36 48 60 72 84 96 108 120 132 144 156 168 180
time
Deaths from cancer

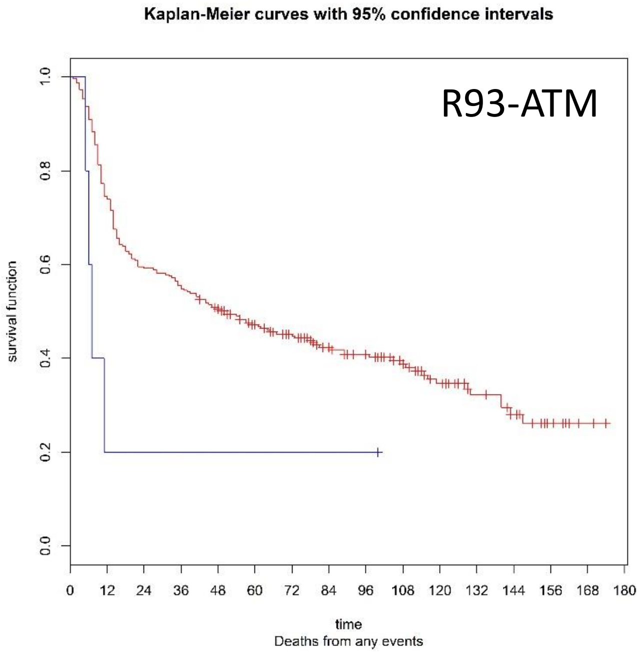
Kaplan-Meier curves with 95% confidence intervals
R93-ATM
survival function
0.0
0.2
0.4
0.6
0.8
1.0
0 12 24 36 48 60 72 84 96 108 120 132 144 156 168 180
time
Deaths from any events

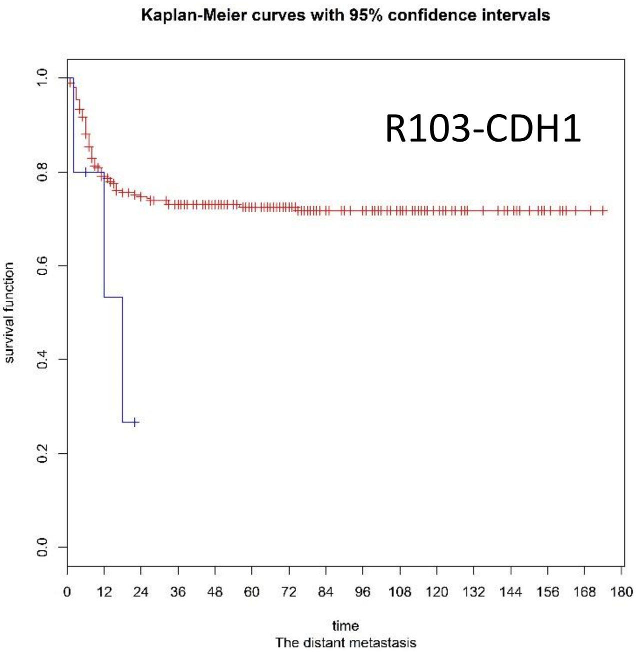
Kaplan-Meier curves with 95% confidence intervals
R103-CDH1
survival function
0.0
0.2
0.4
0.6
0.8
1.0
0 12 24 36 48 60 72 84 96 108 120 132 144 156 168 180
time
The distant metastasis

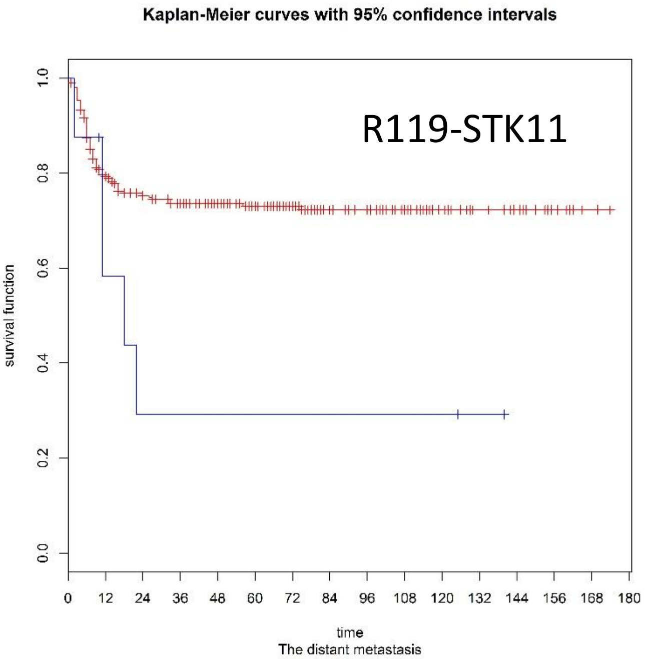
Kaplan-Meier curves with 95% confidence intervals
R119-STK11
survival function
0.0
0.2
0.4
0.6
0.8
1.0
0 12 24 36 48 60 72 84 96 108 120 132 144 156 168 180
time
The distant metastasis

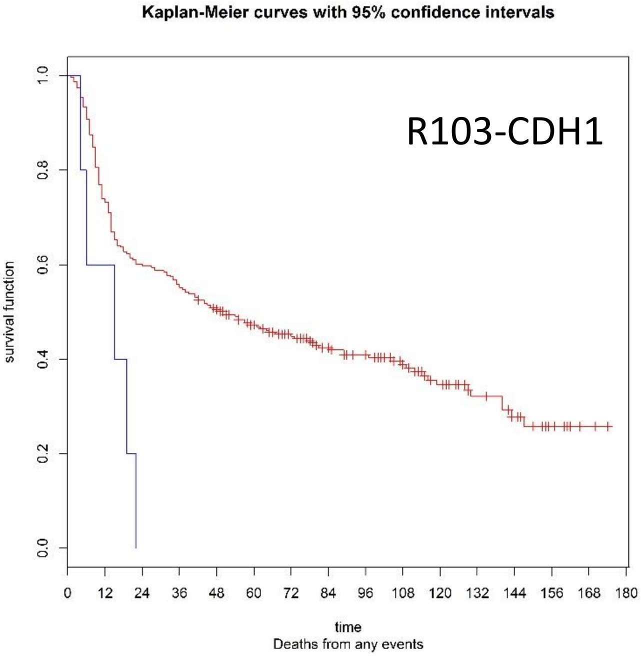
Kaplan-Meier curves with 95% confidence intervals
R103-CDH1
survival function
0.0
0.2
0.4
0.6
0.8
1.0
0 12 24 36 48 60 72 84 96 108 120 132 144 156 168 180
time
Deaths from any events

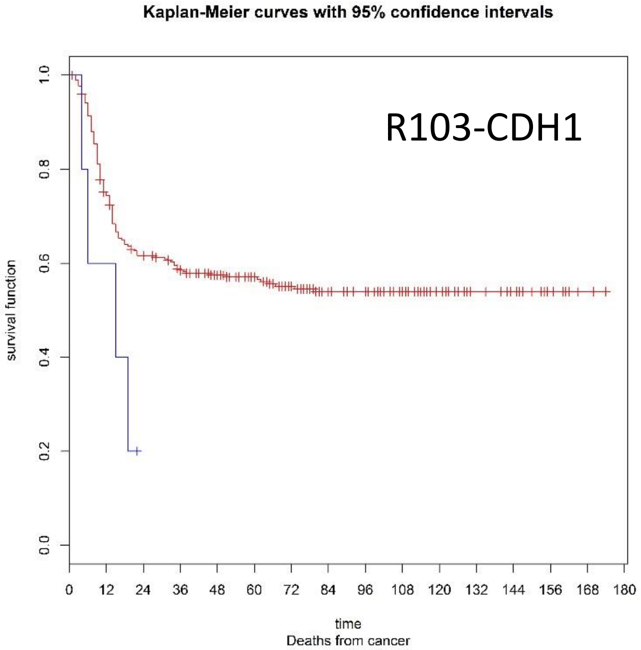
Kaplan-Meier curves with 95% confidence intervals
R103-CDH1
survival function
0.0
0.2
0.4
0.6
0.8
1.0
0 12 24 36 48 60 72 84 96 108 120 132 144 156 168 180
time
Deaths from cancer

Figure S1. Kaplan-Meier plots showing the associations between consistently replicated copy number amplifications and clinical outcomes.

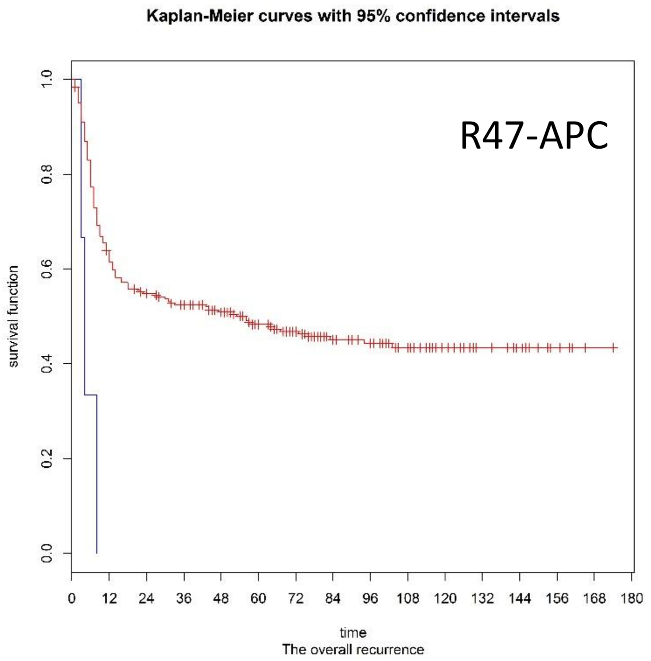
Kaplan-Meier curves with 95% confidence intervals
R47-APC
survival function
0.0 0.2 0.4 0.6 0.8 1.0
0 12 24 36 48 60 72 84 96 108 120 132 144 156 168 180
time
The overall recurrence

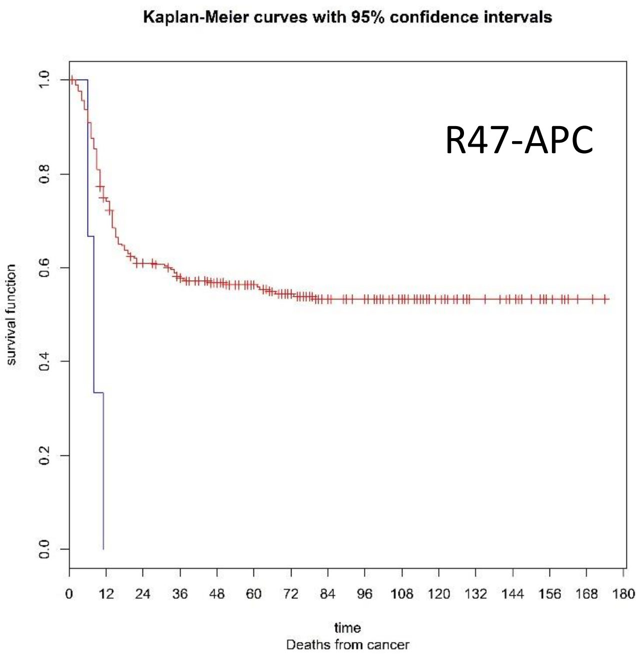
Kaplan-Meier curves with 95% confidence intervals
R47-APC
survival function
0.0 0.2 0.4 0.6 0.8 1.0
0 12 24 36 48 60 72 84 96 108 120 132 144 156 168 180
time
Deaths from cancer

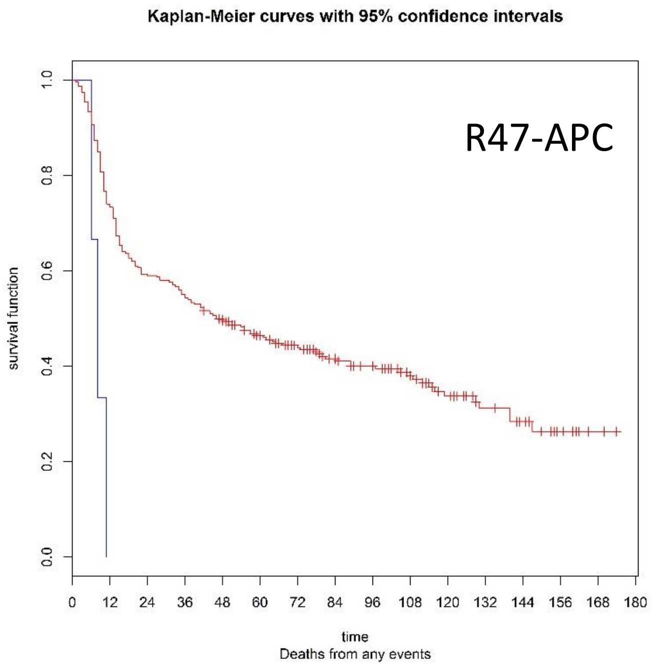
Kaplan-Meier curves with 95% confidence intervals
R47-APC
survival function
0.0 0.2 0.4 0.6 0.8 1.0
0 12 24 36 48 60 72 84 96 108 120 132 144 156 168 180
time
Deaths from any events

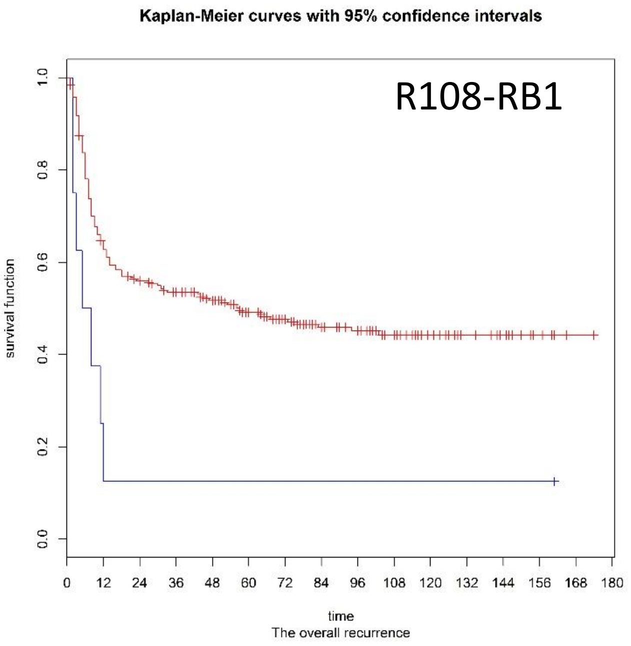
Kaplan-Meier curves with 95% confidence intervals
R108-RB1
survival function
0.0 0.2 0.4 0.6 0.8 1.0
0 12 24 36 48 60 72 84 96 108 120 132 144 156 168 180
time
The overall recurrence

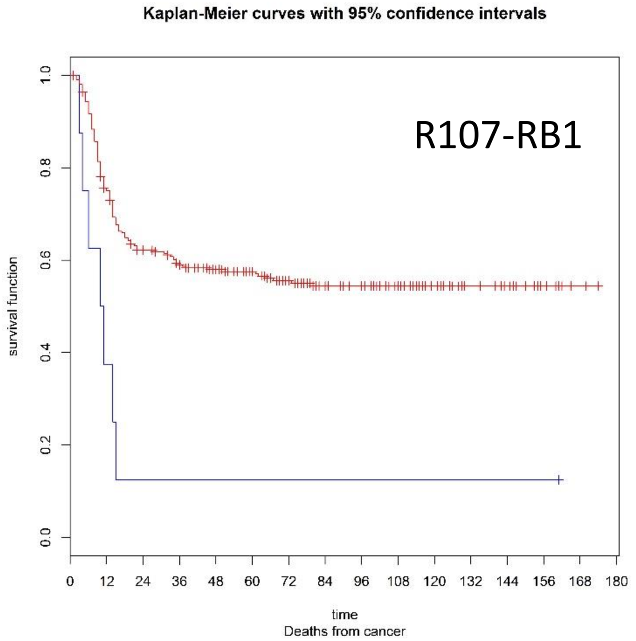
Kaplan-Meier curves with 95% confidence intervals
R107-RB1
survival function
0.0 0.2 0.4 0.6 0.8 1.0
0 12 24 36 48 60 72 84 96 108 120 132 144 156 168 180
time
Deaths from cancer

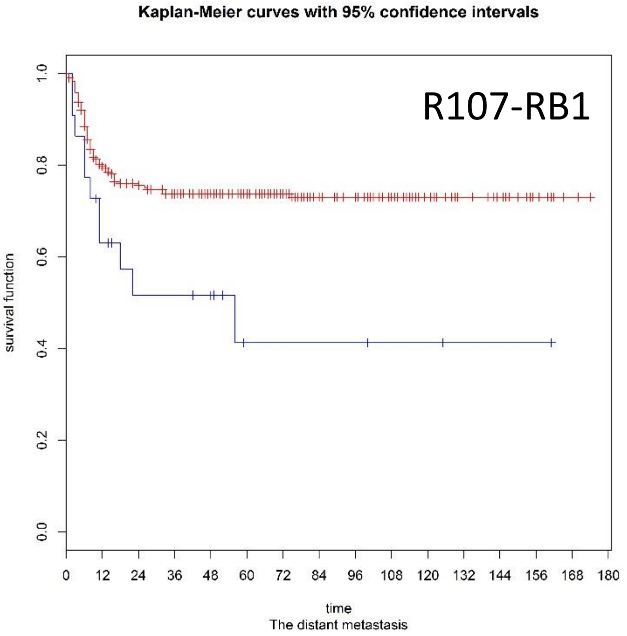
Kaplan-Meier curves with 95% confidence intervals
R107-RB1
survival function
0.0 0.2 0.4 0.6 0.8 1.0
0 12 24 36 48 60 72 84 96 108 120 132 144 156 168 180
time
The distant metastasis

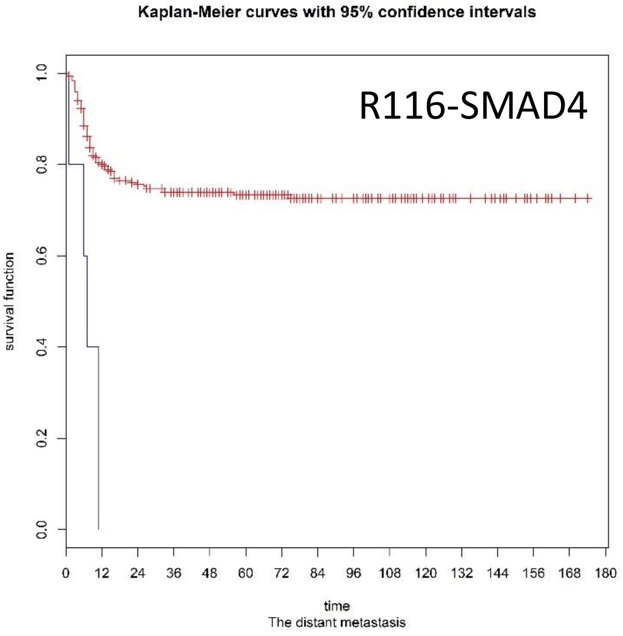
Kaplan-Meier curves with 95% confidence intervals
R116-SMAD4
survival function
1.0
0.8
0.6
0.4
0.2
0.0
0 12 24 36 48 60 72 84 96 108 120 132 144 156 168 180
time
The distant metastasis

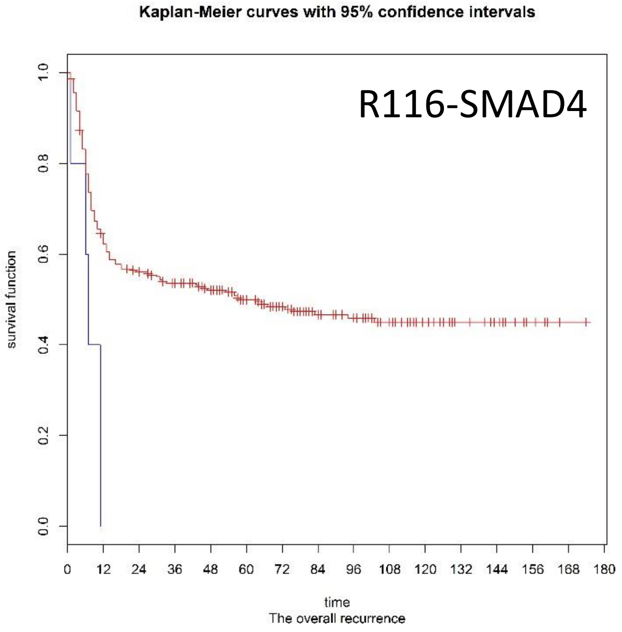
Kaplan-Meier curves with 95% confidence intervals
R116-SMAD4
survival function
1.0
0.8
0.6
0.4
0.2
0.0
0 12 24 36 48 60 72 84 96 108 120 132 144 156 168 180
time
The overall recurrence

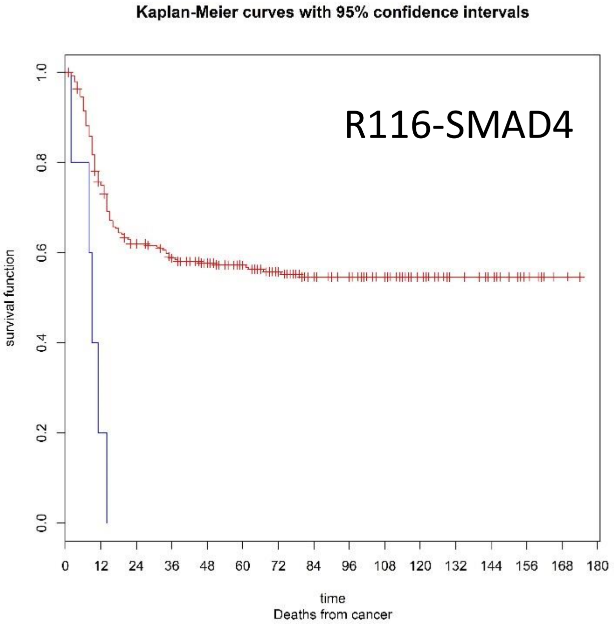
Kaplan-Meier curves with 95% confidence intervals
R116-SMAD4
survival function
1.0
0.8
0.6
0.4
0.2
0.0
0 12 24 36 48 60 72 84 96 108 120 132 144 156 168 180
time
Deaths from cancer

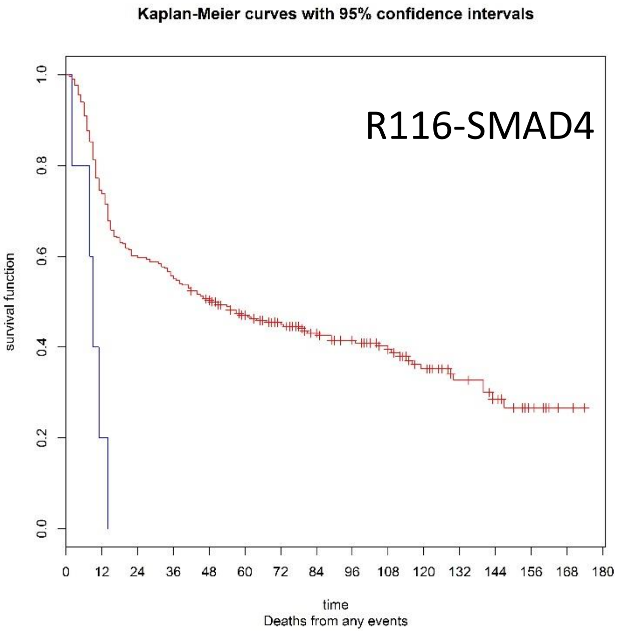
Kaplan-Meier curves with 95% confidence intervals
R116-SMAD4
survival function
1.0
0.8
0.6
0.4
0.2
0.0
0 12 24 36 48 60 72 84 96 108 120 132 144 156 168 180
time
Deaths from any events

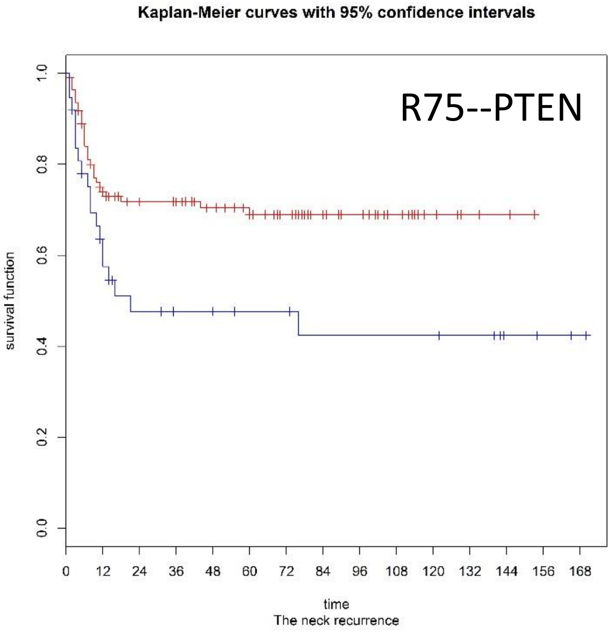
Kaplan-Meier curves with 95% confidence intervals
R75--PTEN
survival function
1.0
0.8
0.6
0.4
0.2
0.0
0 12 24 36 48 60 72 84 96 108 120 132 144 156 168
time
The neck recurrence

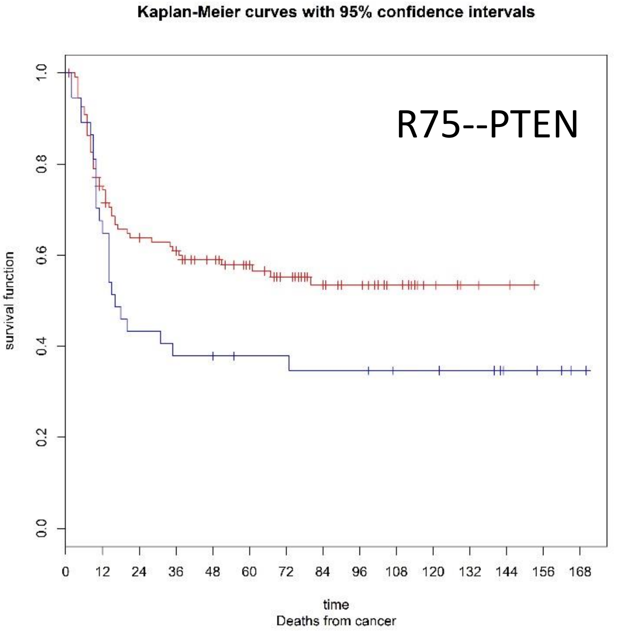
Kaplan-Meier curves with 95% confidence intervals
R75--PTEN
survival function
1.0
0.8
0.6
0.4
0.2
0.0
0 12 24 36 48 60 72 84 96 108 120 132 144 156 168
time
Deaths from cancer

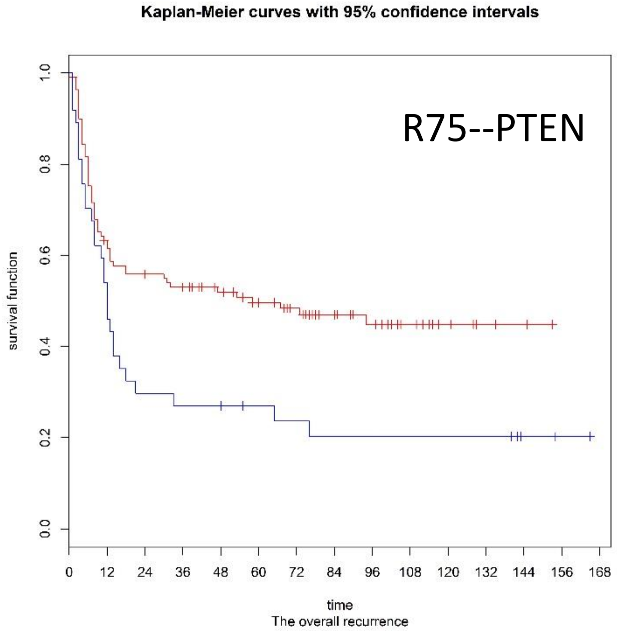

Figure S2. Kaplan-Meier plots showing the associations between consistently replicated copy number deletions and clinical outcomes.

Table S1. List of the 46 genes analyzed by ultra-deep targeted sequencing

ABL1	EGFR	HRAS	NOTCH1	SMARCB1
AKT1	ERBB2	IDH1	NPM1	SMO
ALK	ERBB4	JAK2	NRAS	SRC
APC	FBXW7	JAK3	PDGFRA	STK11
ATM	FGFR1	KDR	PIK3CA	TP53
BRAF	FGFR2	KIT	PTEN	VHL
CDH1	FGFR3	KRAS	PTPN11	
CDKN2A	FLT3	MET	RB1	
CSF1R	GNAS	MLH1	RET	
CTNNB1	HNF1A	MPL	SMAD4	

Table S2. Log-rank tests of different clinical outcomes in relation to focal copy number amplifications

Clinical outcome	CNA-harboring gene	Hazard ratio	FDR adjusted P values
Second primary tumors	ERBB4	5.711	0.05359
Second primary tumors	KDR	4.399	0.128281
Second primary tumors	RB1	3.524	0.11415
Second primary tumors	TP53	11.909	0.000389
Second primary tumors	ERBB2	9.707	0.006449
Local recurrence	PIK3CA	2.332	0.020028
Local recurrence	KIT	3.378	0.116609
Local recurrence	MET	2.147	0.018173
Local recurrence	JAK2	8.834	0.009555
Local recurrence	FGFR2	2.131	0.044413
Local recurrence	ATM	2.302	0.046334
Local recurrence	SMAD4	2.439	0.111215
Local recurrence	GNAS	2.424	0.04367
Local recurrence	SMARCB1	1.712	0.126397
Neck recurrence	ALK	1.720	0.045986
Neck recurrence	PIK3CA	1.793	0.118995

Neck recurrence	FBXW7	0.370	0.059376
Neck recurrence	APC	2.200	0.133036
Neck recurrence	JAK2	6.304	0.00691
Neck recurrence	ATM	24.163	0.006927
Neck recurrence	MPL	0.436	0.076583
Neck recurrence	RB1	8.131	0.01132
Neck recurrence	RET	23.474	0.007416
Distant metastases	MLH1	2.005	0.015232
Distant metastases	PDGFRA	3.285	0.015232
Distant metastases	KDR	2.696	0.086268
Distant metastases	APC	2.986	0.006234
Distant metastases	FGFR1	3.043	0.005267
Distant metastases	FGFR2	0.196	0.043997
Distant metastases	HRAS	1.881	0.095354
Distant metastases	FLT3	0.253	0.136246
Distant metastases	CDH1	3.182	0.125833
Distant metastases	ERBB2	3.535	0.086268
Distant metastases	STK11	66.444	0.000397
Distant metastases	GNAS	1.904	0.137368
Distant metastases	SMO	8.034	0.011414
Distant metastases	TP53	7.232	0.000422

Distant metastases	FGFR3	5.273	0.001057
Distant metastases	ERBB2	2.816	0.112271
Distant metastases	HNF1A	2.694	0.021986
Distant metastases	RB1	9.043	0.00683
Distant metastases	RET	13.325	0.031506
Disease-free survival	PIK3CA	1.987	0.002668
Disease-free survival	FBXW7	0.597	0.108512
Disease-free survival	APC	2.644	0.001777
Disease-free survival	MET	1.775	0.005425
Disease-free survival	JAK2	5.516	0.00285
Disease-free survival	ATM	14.102	0.027695
Disease-free survival	HRAS	1.814	0.024415
Disease-free survival	ERBB2	2.980	0.088398
Disease-free survival	SMO	4.069	0.128539
Disease-free survival	PDGFRA	31.941	0.00285
Disease-free survival	FGFR3	3.077	0.021433
Disease-free survival	STK11	31.098	0.003045
Disease-free survival	RB1	4.674	0.087732
Disease-free survival	RET	14.376	0.026579
Disease-specific survival	PIK3CA	1.945	0.006481
Disease-specific survival	MLH1	1.538	0.086341

Disease-specific survival	FBXW7	0.530	0.061604
Disease-specific survival	APC	2.655	0.002528
Disease-specific survival	MET	2.885	0.003454
Disease-specific survival	FGFR1	2.184	0.035701
Disease-specific survival	ABL1	1.546	0.067327
Disease-specific survival	JAK2	7.222	0.000383
Disease-specific survival	ATM	12.804	0.034817
Disease-specific survival	HRAS	1.738	0.057376
Disease-specific survival	CDH1	2.781	0.101434
Disease-specific survival	SMAD4	0.258	0.126938
Disease-specific survival	SMO	4.711	0.07245
Disease-specific survival	PDGFRA	31.674	0.00289
Disease-specific survival	TP53	4.274	0.09712
Disease-specific survival	FGFR3	3.009	0.042483
Disease-specific survival	STK11	31.244	0.002974
Disease-specific survival	ERBB2	2.658	0.050597
Disease-specific survival	RB1	5.431	0.047742
Disease-specific survival	RET	9.033	0.07245
Overall survival	PIK3CA	1.697	0.020329
Overall survival	FBXW7	1.724	0.050968
Overall survival	KDR	1.873	0.073175

Overall survival	APC	2.091	0.022218
Overall survival	MET	1.481	0.064844
Overall survival	JAK2	6.659	0.000668
Overall survival	ATM	11.899	0.0401
Overall survival	CDH1	3.323	0.023086
Overall survival	ERBB2	2.622	0.129804
Overall survival	SMAD4	0.322	0.064816
Overall survival	SMO	4.391	0.091564
Overall survival	PDGFRA	27.164	0.004311
Overall survival	TP53	3.982	0.122979
Overall survival	FGFR3	3.043	0.020864
Overall survival	STK11	27.273	0.004311
Overall survival	RB1	5.139	0.056585
Overall survival	RET	8.475	0.085023

Table S3. Log-rank tests of different clinical outcomes in relation to focal copy number deletions

Clinical outcome	CNA-harboring gene	Hazard ratio	FDR adjusted P values
Second primary tumors	ERBB4	9.363	0.115586
Second primary tumors	SMAD4	7.590	0.193956
Local recurrence	KIT	0.571	0.177049
Local recurrence	EGFR	3.220	0.163241
Local recurrence	ATM	0.615	0.17247
Local recurrence	HNF1A	9.266	0.099714
Local recurrence	RB1	4.998	0.007106
Local recurrence	SMO	2.438	0.092747
Local recurrence	PTEN	0.546	0.118786
Local recurrence	ERBB2	9.561	0.093865
Local recurrence	RET	2.303	0.123509
Neck recurrence	ERBB4	2.013	0.114452
Neck recurrence	APC	9.742	0.000499
Neck recurrence	SMAD4	16.956	5.06E-05
Neck recurrence	KIT	0.580	0.107829
Neck recurrence	TP53	3.277	0.05301
Neck recurrence	RB1	2.451	0.040416

Neck recurrence	BRAF	0.602	0.12575
Neck recurrence	PTEN	0.403	0.110695
Neck recurrence	PDGFRA	3.863	0.054354
Distant metastases	PTEN	66.158	0.000425
Distant metastases	RB1	2.270	0.039665
Distant metastases	TP53	2.025	0.0600823
Distant metastases	SMO	4.606	0.110999
Distant metastases	SMAD4	11.594	0.000123
Disease-free survival	ERBB4	1.900	0.049063
Disease-free survival	PDGFRA	3.646	0.036772
Disease-free survival	APC	5.702	0.011237
Disease-free survival	MET	4.128	0.144504
Disease-free survival	PTEN	31.155	0.003559
Disease-free survival	RB1	3.021	0.014776
Disease-free survival	SMAD4	5.556	6.89E-05
Disease-specific survival	ERBB4	1.872	0.086112
Disease-specific survival	KIT	0.651	0.092464
Disease-specific survival	APC	6.071	0.008054
Disease-specific survival	MET	5.472	0.050529
Disease-specific survival	ABL1	1.942	0.13819
Disease-specific survival	PTEN	31.532	0.003566

Disease-specific survival	RB1	3.600	0.003651
Disease-specific survival	SMAD4	5.426	0.00093
Overall survival	KIT	0.674	0.053378
Overall survival	APC	5.794	0.009984
Overall survival	MET	5.214	0.05303
Overall survival	PTEN	27.218	0.005174
Overall survival	RB1	2.558	0.049577
Overall survival	SMAD4	5.144	0.001345

Table S4 Pathogenic significance of genes with validated CNA in different human malignancies according to the published literature

Gene	Associated malignancy	
ABL1	Acute lymphoblastic leukemia	[1]
FGFR1	Oral squamous cell carcinoma	[2]
FLT3	Cutaneous myelomonocytic leukemia	[3]
KDR	Glioblastoma, primitive neuroectodermal tumors	[4-6]
KIT	Glioblastoma, primitive neuroectodermal tumors	[4-6]
PIK3CA	Oral squamous cell carcinoma	[7]
RB1	Oral squamous cell carcinoma,<br>breast cancer,<br>chronic lymphocytic leukemia,<br>retinoblastoma	[8-11]
RET	Thyroid carcinoma	[12,13]
SMO	Melanoma	cBIO, 2014 (http://www.cbioportal.org/ctd2-dashboard-portal/index.do)
APC	Colorectal cancer, gastric adenocarcinomas	[14,15]
EGFR	Small cell lung cancer, non-small cell lung cancer	[16,17]
MET	Small cell lung cancer, non-small cell lung cancer	[18,19]
PTEN	Oral squamous cell carcinoma,	[20,21]

	prostate adenocarcinomas	
SMAD4	Oral squamous cell carcinoma	[22]
TP53	Oral squamous cell carcinoma	[9,23,24]

1. Bernasconi P, Calatroni S, Giardini I, Inzoli A, Castagnola C, et al. (2005) ABL1 amplification in T-cell acute lymphoblastic leukemia. Cancer Genet Cytogenet 162: 146-150.
2. Freier K, Schwaenen C, Sticht C, Flechtenmacher C, Muhling J, et al. (2007) Recurrent FGFR1 amplification and high FGFR1 protein expression in oral squamous cell carcinoma (OSCC). Oral Oncol 43: 60-66.
3. Dijkman R, van Doorn R, Szuhai K, Willemze R, Vermeer MH, et al. (2007) Gene-expression profiling and array-based CGH classify CD4+CD56+ hematodermic neoplasm and cutaneous myelomonocytic leukemia as distinct disease entities. Blood 109: 1720-1727.
4. Nobusawa S, Stawski R, Kim YH, Nakazato Y, Ohgaki H (2011) Amplification of the PDGFRA, KIT and KDR genes in glioblastoma: a population-based study. Neuropathology 31: 583-588.
5. Holtkamp N, Ziegenhagen N, Malzer E, Hartmann C, Giese A, et al. (2007) Characterization of the amplicon on chromosomal segment 4q12 in glioblastoma multiforme. Neuro Oncol 9: 291-297.
6. Joensuu H, Puputti M, Sihto H, Tynninen O, Nupponen NN (2005) Amplification of genes encoding KIT, PDGFRalpha and VEGFR2 receptor tyrosine kinases is frequent in glioblastoma multiforme. J Pathol 207: 224-231.
7. Murugan AK, Munirajan AK, Tsuchida N (2013) Genetic deregulation of the PIK3CA oncogene in oral cancer. Cancer Lett 338: 193-203.
8. Chao HH, He X, Parker JS, Zhao W, Perou CM (2012) Micro-scale genomic DNA copy number aberrations as another means of mutagenesis in breast cancer. PLoS One 7: e51719.
9. Choi S, Myers JN (2008) Molecular pathogenesis of oral squamous cell carcinoma: implications for therapy. J Dent Res 87: 14-32.
10. Ouillette P, Collins R, Shakhan S, Li J, Li C, et al. (2011) The prognostic significance of various 13q14 deletions in chronic lymphocytic leukemia. Clin Cancer Res 17: 6778-6790.
11. Castera L, Dehainault C, Michaux D, Lumbroso-Le Rouic L, Aerts I, et al. (2013) Fine mapping of whole RB1 gene deletions in retinoblastoma patients confirms PCDH8 as a candidate gene for psychomotor delay. Eur J Hum Genet 21: 460-464.
12. Nakashima M, Takamura N, Namba H, Saenko V, Meirmanov S, et al. (2007) RET oncogene amplification in thyroid cancer: correlations with radiation-associated and high-grade malignancy. Hum Pathol 38: 621-628.
13. Ciampi R, Romei C, Cosci B, Vivaldi A, Bottici V, et al. (2012) Chromosome 10 and RET gene copy number alterations in hereditary and sporadic Medullary Thyroid Carcinoma. Mol Cell Endocrinol 348: 176-182.
14. Fang Z, Xiong Y, Li J, Liu L, Zhang W, et al. (2012) APC gene deletions in gastric adenocarcinomas in a Chinese population: a correlation with tumour progression. Clin Transl Oncol 14: 60-65.
15. Bogaert J, Prenen H (2014) Molecular genetics of colorectal cancer. Ann Gastroenterol 27: 9-14.
16. Rosell R, Taron M, Reguart N, Isla D, Moran T (2006) Epidermal growth factor receptor activation: how exon 19 and 21 mutations changed our understanding of the pathway. Clin Cancer Res 12: 7222-7231.
17. Marchetti A, Del Grammastro M, Filice G, Felicioni L, Rossi G, et al. (2012) Complex mutations &

subpopulations of deletions at exon 19 of EGFR in NSCLC revealed by next generation sequencing: potential clinical implications. PLoS One 7: e42164.
18. Kong-Beltran M, Seshagiri S, Zha J, Zhu W, Bhawe K, et al. (2006) Somatic mutations lead to an oncogenic deletion of met in lung cancer. Cancer Res 66: 283-289.
19. Beau-Faller M, Ruppert AM, Voegeli AC, Neuville A, Meyer N, et al. (2008) MET gene copy number in non-small cell lung cancer: molecular analysis in a targeted tyrosine kinase inhibitor naive cohort. J Thorac Oncol 3: 331-339.
20. Wang SI, Parsons R, Ittmann M (1998) Homozygous deletion of the PTEN tumor suppressor gene in a subset of prostate adenocarcinomas. Clin Cancer Res 4: 811-815.
21. Mendes RA (2012) Oncogenic Pathways in the Development of Oral Cancer. Journal of Carcinogenesis & Mutagenesis 3:2.
22. Wang X, Sun W, Bai J, Ma L, Yu Y, et al. (2009) Growth inhibition induced by transforming growth factor-beta1 in human oral squamous cell carcinoma. Mol Biol Rep 36: 861-869.
23. Stoicanescu D, Andreescu N, Belengeanu A, Meszaros N, Cornianu M (2013) Assessment of p53 and HER-2/neu genes status and protein products in oral squamous cell carcinomas. Rom J Morphol Embryol 54: 1107-1113.
24. Wong YK, Liu TY, Chang KW, Lin SC, Chao TW, et al. (1998) p53 alterations in betel quid- and tobacco-associated oral squamous cell carcinomas from Taiwan. J Oral Pathol Med 27: 243-248.
